# Detection of PCBs and OCPs in the Irtysh River Water (GC-MS/MS) and ecological risk assessment

**DOI:** 10.1016/j.mex.2024.102944

**Published:** 2024-09-02

**Authors:** Shi-Zhan Tang, Zhong-Xiang Chen, Qi-Rui Hao, Yao-Peng Hu, Ji-Long Wang, Dong-Li Qin, Peng Wang, Hai-Tao Wang

**Affiliations:** a Heilongjiang River Fisheries Research Institute, Chinese Academy of Fishery Sciences, Harbin 150070, China; b Heilongjiang River Basin Fishery Ecological Environment Monitoring Center, Ministry of Agriculture and Rural Affairs, Harbin 150070, China

**Keywords:** Irtysh river, PCBs, OCPs, GC-MS/MS, Ecological risk, Assessment, Gas chromatography-triple quadrupole mass spectrometer (GC-MS/MS);Ecological Risk Assessment Methods

## Abstract

This study optimized a gas chromatography-tandem triple quadrupole mass spectrometry (GC-MS/MS) method for the determination of 21 persistent organic pollutants (POPs) in Irtysh River water, including 14 organochlorines (OCPs) and 7 polychlorinated biphenyls (PCBs). Factors such as column temperature ramping, selection of qualitative and quantitative ion pairs and collision energy were considered to achieve perfect separation and accurate quantification of all 21 target compounds. The limits of detection (LOD) for PCBs and OCPs ranged from 0.21 to 1.18 ng/L. Applying this method to detect POPs in the Irtysh River revealed concentrations of OCPs ranging from ND to 20.2 ng/L and PCBs from ND to 0.411 ng/L. Source analysis indicated that POPs in the Irtysh River mainly originate from historical industrial and agricultural activities, particularly the deliberate use of pesticides. To ensure ecological safety and human health, expanding the range of target analytes and monitoring periods is necessary. This study provides:

• Qualitative and quantitative analysis methods for 7 PCBs and 14 OCPs.
• Recoveries achieved ranged between 74.6 to 109 % with RSD less than 15 %.
• Analysis of sources, transport pathways, accumulation status, and ecological risks of PCBs and OCPs in the Irtysh River.

## Graphical abstract

**Figure:**
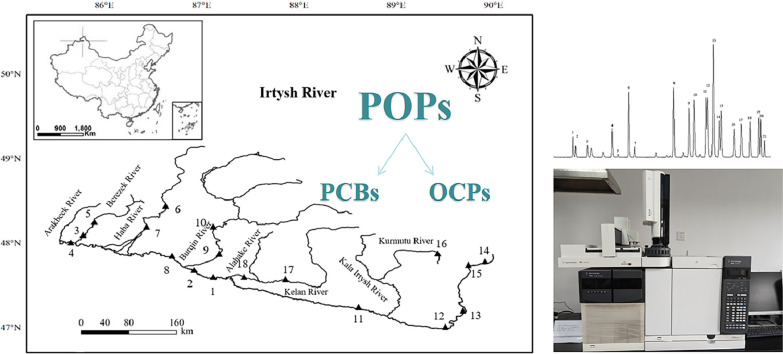

Specifications table

**Table:** 

Subject area:	Environmental Science
More specific subject area:	Environmental pollutant monitoring
Name of your method:	Gas chromatography-triple quadrupole mass spectrometer (GC-MS/MS); Ecological Risk Assessment Methods
Name and reference of original method:	Sohail, M., Eqani, S., Bokhari, H., Hashmi, M.Z., Ali, N., Alamdar, A., Podgorski, J.E., Adelman, D., Lohmann, R. (2022) Freely Dissolved Organochlorine Pesticides (OCPs) and Polychlorinated Biphenyls (PCBs) along the Indus River Pakistan: Spatial pattern and Risk Assessment. Environmental Science and Pollution Research. 29(43), 65670-65683.
Resource availability:	None

## Background

The Irtysh River not only serves as a major source of domestic water and irrigation water for local residents, but also serves as an important habitat for plenty of native fish and other aquatic organisms. It has played a significant role in maintaining the balance of water ecology and in stabilizing the water environment [[1], [2], [3]]. However, in recent decades, human activities have brought pressure on the ecosystem of Irtysh River. Especially, land reclamation, agricultural irrigation, animal husbandry and climate change have caused Irtysh River's environment to fluctuate greatly in the past decades. This situation will pose a threat to the regional ecology and international relations, deteriorate the water quality, reduce habitats and tension the relations between the upstream countries and the downstream countries [[4], [5]].

PCBs and OCPs are ubiquitous anthropogenic pollutants characterized by their high toxicity, long-range transport, bioaccumulation, and biomagnification in ecosystems. They have long half-lives in sediments, water and organisms, which can potentially pose risks to the ecological environment and human health [[6], [7]]. Therefore, the PCBs and OCPs residues in water environment have raised increasing concern worldwide [[8], [9]]. Though most organochlorine compounds were prohibited for many years in the last century, certain levels of them can still be detected in many waters and sediments both at home and abroad. For example, PCBs have been detected in lake sediments in Tibet, China. In certain natural water systems in Asia and Africa, OCPs have detection rates of 93% and 100%, respectively. In Europe and North America, the average concentration of hexachlorobenzene is 1 ng/L. [[10], [11], [12]]. At present, there is still a lack of evaluations and researches on the ecological risks of PCBs and OCPs in Irtysh River.

Currently, there are basically three ways to test the organochlorine pesticides contained in the environment, including gas chromatographic method (GC) [13], gas chromatography – mass spectrometry (GC-MS) [[14], [15]] and gas chromatography - tandem mass spectrometry (GC-MS/MS) [16] respectively. GC-MS/MS features a strong anti-interference, a high sensitivity, a great selectivity and so on. At present, most studies of this kind focus on the separate determination of PCBs and OCPs residues in waters or sediments. There are few studies describing the simultaneous determination of PCBs and OCPs residues in water samples by GC-MS/MS [[13], [14], [15], [16]].

In response, we have developed a GC-MS/MS method to detect the levels and distribution of POPs (Persistent Organic Pollutants) in the Irtysh River. We have provided sample collection methods, sample pre-treatment procedures, and optimized instrument and analysis conditions. These efforts aim to offer readers reliable detection techniques and valuable scientific data references.

## Method details

### Instruments and reagents

The instruments used in this study included the 7000C-7890B gas chromatography – tandem mass spectrometer (America's Agilent) equipped with the electron impact (EI) ion source, XS205 Dual Range electronic analytical balance (produced by Switzerland's Mettler Toledo, with its sensitivity being 0.01 mg), Allegra X-30R high-speed centrifuge (America's Beckman), microwave muffle furnace (America's THERMO), N-EVAP112 sample concentrator (China's LWL), and MilliQ ultrapure water machine (America's Millipore).

The mixed standard solution concentration of the 7 PCBs and 14 OCPs was 100 mg/L (Tianjin-based Alta Scientific Co., Ltd.). The normal hexane, acetone, methylene chloride and acetonitrile were chromatographic pure reagents (America's J. T. Baker). The concentration of Florey silica solid-phase extraction column, amino solid-phase extraction column and silica gel solid-phase extraction column was 500 mg / 3 mL (ANPEL Laboratory Technologies (Shanghai) Inc.).

### Preparation of standard solution

The mixed standard solution of 7 PCBs and 14 OCPs was diluted by 100 times to obtain the intermediate stock solution which was then respectively made with isooctane into the mixed standard solutions with the mass concentrations of 1, 5, 10, 20, 50 and 100 µg/L, for later use.

### Analysis conditions of instrument

Gas phase condition: Chromatographic column: HP-5MS (30 m × 0.25 mm × 0.25 µm); Injection port temperature: 290°C; Injection capacity: 1 µL; Carrier gas: High-purity helium (≥99.999%); Flow rate: 1.0 mL/min; Injection mode: Splitless injection. Turn on the side valve and washer purging valve 1.0 min later, with the purging flow rate being 30 mL/min. Turn on the carrier gas saver 2 min later, with the flow rate being 20 mL/min. Chromatographic column temperature rise procedures: Keep the initial temperature at 80°C for 1min, raise the temperature to 170°C at the rate of 30°C/min, and then raise the temperature to 240°C at the rate of 5°C/min and keep it for 1min. At last, raise the temperature to 300°C at the rate of 20°C/min, and keep it for 3 min.

Mass spectrometry conditions: The electron impact (EI) ion source was used. Ionization energy: 70 eV; Ion source temperature: 300°C; Temperature of mass spectral transmission line: 280°C; Data collection mode: Selected reaction monitoring (SRM); Collision gas: High-purity helium (≥99.999%); Solvent delay time: 8.00 min.

### Health risk evaluation

In this study, the quotient method based on the EPA standard was used to evaluate the ecological risks of the OCPs and PCBs contained in the water of Irtysh River, with the formula shown as follows [17]:

(1) $$Q=\frac{MEC}{\text{MSNOCE}}$$

In this formula, Q refers to the quotient of risk value, MEC (measured environment concentration) is the tested concentration of target compound, and MSNOCE (multi-species no observable effect concentration) is the no observable effect content of target compound. According to the characterization principle of the quotient method, it means no ecological risk if Q is less than 1, a low ecological risk if Q is equal to or greater than 1 but less than 5, a medium risk if Q is equal to or greater than 5 but less than 10, and a high risk if Q is equal to or greater than 10.

## Results and discussion

**Fig. 1:**
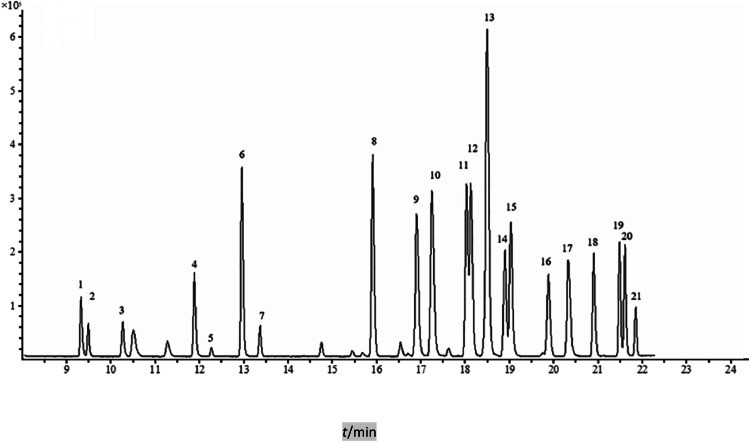
Total Ion Chromatography (TIC) of 7 PCBs and 14 OCPs.

Perform a full scan of the mixed standard solution at a concentration of 200 ng/mL, which contains 7 PCBs and 14 OCPs. This scan will provide the maximum mass-to-charge ratios (m/z) for 21 target compounds, thereby clarifying the parent ion information. Then the product ion method was used to optimize the collision energy and to determine the quantitative and the qualitative ion. Finally, the SRM method was constructed based on the retention time of the 21 target samples, the parent ion, m/z of daughter ion, the collision energy and so on. The mass spectrum parameters and detailed information for the target compounds are shown in Table 1 and Table S1, and the total ion chromatography of the 7 PCBs and the 14 OCPs are shown in Fig. 1.

**Table 1:** SRM Conditions, Linear Ranges, Correlation Coefficients and Limits of Detection (LOD) of 7 PCBs and 14 OCPs (n=7).

Analyte	Retention time / min	Monitoring ion-pair / (m /z)	Collision energy /(eV)	Linear range / (μg/L)	R^2^	LOD
α-HCH	9.440	218.8 / 183.0	10	1—100	0.9992	0.46
		182.8 / 146.7	16			
Hexachlorobenzene	9.598	283.8 / 213.8	30	1—100	0.9992	0.87
		283.8 / 248.8	18			
β-HCH	10.374	218.7 / 183.0	10	1—100	0.9998	0.99
		180.9 / 145.0	16			
γ-HCH	11.974	218.7 / 183.0	10	1—100	0.9993	0.21
		180.9 / 145.0	16			
δ-HCH	12.355	218.8 / 182.9	10	1—100	0.9994	1.02
		182.8 / 146.7	16			
PCB 28	13.041	256.0 / 186.0	26	1—100	0.9996	0.30
		258.0 / 186.0	26			
Heptachlor	13.450	271.8 / 236.9	12	1—100	0.9987	0.94
		99.8 / 65.0	26			
PCB 52	15.901	220.0 / 150.0	26	1—100	0.9994	0.96
		255.0 / 220.0	26			
Aldrin	16.946	262.7 / 192.9	32	1—100	0.9999	1.12
		262.7 / 191.0	30			
Heptachlor epoxide	17.332	252.8 / 262.9	16	1—100	0.9995	0.53
		354.7 / 264.9	12			
PCB 101	18.167	323.9 / 254.0	24	1—100	0.9993	0.57
		326.0 / 256.0	22			
p,p_-DDE	18.531	246.0 / 176.1	28	1—100	0.9972	1.18
		317.8 / 246.0	20			
Dieldrin	18.660	262.8 / 192.9	30	1—100	0.9995	1.18
		262.8 / 190.9	30			
Endrin	18.805	262.8 / 192.9	30	1—100	0.9984	0.37
		245.0 / 173.0	22			
PCB 118	19.174	323.9 / 254.0	26	1—100	0.9977	0.93
		326.0 / 256.0	26			
p,p_-DDD	19.822	235.0 / 165.1	20	1—100	0.9997	0.60
		236.8 / 165.0	20			
o,p_-DDT	20.405	235.0 / 165.1	20	1—100	0.9995	1.01
		236.8 / 165.0	20			
PCB 153	20.911	290.0 / 220.0	28	1—100	0.9996	0.74
		359.9 / 289.9	28			
p,p_-DDT	21.531	235.0 / 165.1	22	1—100	0.9997	0.90
		236.8 / 165.0	22			
PCB 138	21.645	290.0 / 220.0	24	1—100	0.9996	0.60
		359.9 / 289.9	24			
PCB 180	21.862	393.9 / 323.8	28	1—100	0.9977	0.75
		395.9 / 323.8	28

1. α-HCH; 2. Hexachlorobenzene; 3. β-HCH; 4. γ-HCH; 5. δ-HCH; 6. PCB 28; 7. Heptachlor; 8. PCB 52; 9. Aldrin; 10. Heptachlor epoxide; 11. PCB 101; 12. p,p_-DDE; 13. Dieldrin; 14. Endrin; 15. PCB 118; 16. p,p_-DDD; 17. o,p_-DDT; 18. PCB 153; 19. p,p_-DDT; 20. PCB 138; 21. PCB 180.

### Quality assurance/quality control (QA/QC)

Under the best test condition determined, the mixed standard solutions with the mass concentrations of 1, 5, 10, 20, 50 and 100 µg/L respectively, were tested. A standard curve was made based on the regression analysis on the mass concentration of standard solution with the response intensity of 7 PCBs and 14 OCPs, with the related coefficients ranging from 0.9972 to 0.9999. Quantification was performed using the external standard method with a standard curve, with blank values subtracted and the results multiplied by the dilution factor to determine the concentration of the target substances in the samples. Add the mixed standard solution of 7 PCBs and 14 OCPs with a concentration near the limits of detection to the blank water samples that did not contain the target compounds, and then make analysis on it. Work out the standard deviation (SD) according to the 7 parallel samples, and then work out the limits of detection (LOD) based on the formula LOD = t × SD, when the number of replicate measurements is 7 and the confidence level is 99%, the t-value is 3.143 [18]. The limits of detection for the substances of the River ranged from 0.21 to 1.18 ng/L. The linear ranges, R^2^ and LOD were shown in Table 1.

The spiked recovery was adopted in this study. Add the mixed standard solutions of the low concentration, medium concentration and high concentration to the blank water sample. Test the sample three times by following the experimental method. The average spiked recoveries and the relative standard deviations of 7 PCBs and 14 OCPs in the aquatic water were shown in Table 2. It can be seen from Table 2 that the average recoveries of 7 PCBs and 14 OCPs in waters ranged from 74.6% to 109%, and the RSD ranged from 2.50% to 13.9%. Therefore, it met the test requirement.

**Table 2:** Spiked Recoveries and Relative Standard Deviations of 7 PCBs and 14 OCPs in the Aquatic Water (n=3).

Analyte	Low level			Medium level			High level		
	Spikedng/L	Recovery %	RSD%	Spikedng/L	Recovery %	RSD%	Spikedng/L	Recovery %	RSD%
α-HCH	10	80.9	10.3	50	94.0	10.9	100	97.1	6.22
δ-HCH	10	74.6	4.53	50	90.4	7.41	100	96.1	8.50
β-HCH	10	89.2	8.20	50	86.6	10.2	100	89.4	6.47
Hexachlorobenzene	10	92.9	11.0	50	87.7	9.74	100	102	5.81
γ-HCH	10	98.2	3.74	50	90.5	3.32	100	99.3	6.10
PCB 28	10	90.1	6.53	50	90.9	2.50	100	104	9.18
Heptachlor	10	98.3	4.20	50	91.4	5.70	100	95.4	5.30
PCB 138	10	89.4	9.64	50	88.1	8.38	100	100	7.51
PCB 153	10	98.5	5.28	50	95.2	5.90	100	98.5	8.90
PCB 52	10	89.1	9.21	50	87.7	7.07	100	99.2	8.43
Aldrin	10	92.3	4.04	50	98.3	9.70	100	88.8	7.78
Dieldrin	10	109	5.03	50	87.1	5.52	100	95.4	6.82
Endrin	10	81.8	10.2	50	86.0	6.10	100	90.7	3.00
Heptachlor epoxide	10	108	6.62	50	101	10.6	100	88.7	6.67
p,p_-DDE	10	91.7	5.80	50	89.5	8.23	100	89.8	10.0
PCB 118	10	100	13.9	50	96.2	8.22	100	104	8.00
PCB 101	10	90.4	8.50	50	85.0	7.33	100	92.1	7.02
o,p_-DDT	10	88.2	9.83	50	95.5	3.01	100	87.3	9.77
p,p_-DDD	10	86.8	8.77	50	97.8	6.26	100	84.5	6.74
p,p_-DDT	10	103	5.40	50	96.2	6.50	100	87.7	3.08
PCB 180	10	104	9.51	50	95.0	7.98	100	94.3	5.30

### Additional information

**Fig. 2:**
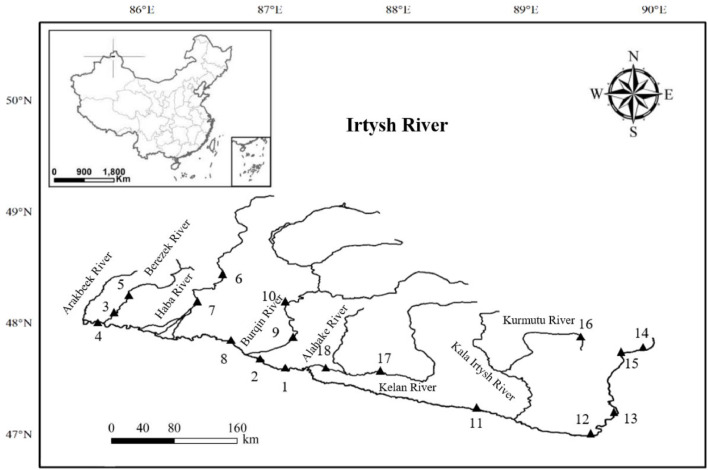
Map of Sampling Sites in the Irtysh River.

In this study, this test method was used to analyze the Irtysh water samples in May 2021. A total of 18 water samples were collected (Fig. 2), including 9 from the typical trunk stream areas and 9 from its major tributaries. At each sampling site, 10 L of surface water (20 cm below the water surface) was sampled. Take 500 mL of the well-mixed water sample, and place it in a 1.0 L separating funnel. Add 20.0 mL of methylene chloride to the funnel for liquid-liquid extraction, and leave the mixture for layering. Take the extract liquor from the lower layer, and repeat the extraction once. Merge the extract liquor in the 50 mL brown glass vials, and seal them. Timely deliver these vials to the lab, and store them at 4°C for further analysis.

Sample pretreatment: The extract liquor was purified by the concentrated sulphuric acid until become colorless, and its organic phase was washed to neutral. The extract was gradually transferred to a 150 mL heart-shaped bottle and evaporated to near dryness at 40°C, after being dehydrated by the anhydrous sodium sulphate. Take proper amount of dissolved hexane residue, fill its volume to 1.00 mL, and then leave it on the instrument to be tested. The ultrapure water was selected as the blank control sample which was treated with the same method.

From Table 3, we can find out that among all the water samples, only certain levels of OCPs, including α-HCH, δ-HCH, β-HCH, γ-HCH, PCB 28, Aldrin, Dieldrin and Endrin, were detected, with the detection rate ranging from 5.55 to 27.8%. The detection ranges of these OCPs were α-HCH: ND - 8.23 ng/L, δ-HCH: ND - 6.74 ng/L, β-HCH: ND - 5.27 ng/L, γ-HCH: ND - 0.332 ng/L, PCB 28: ND - 0.411 ng/L, Aldrin: ND - 3.73 ng/L, Dieldrin: ND - 4.86 ng/L and Endrin: ND - 6.15 ng/L respectively.

**Table 3:** Status of PCBs and OCPs Pollution in the Irtysh River.

Analyte	CAS	Min (ng/L)	Max (ng/L)	Average (ng/L)	SD (ng/L)	Detection rate (%)
α-HCH	319-84-6	N.D	8.23	1.48	2.94	27.8
δ-HCH	319-86-8	N.D	6.74	0.916	2.22	16.7
β-HCH	319-85-7	N.D	5.27	0.836	1.81	22.2
Hexachlorobenzene	118-74-1	N.D	N.D	N.D	N.D	N.D
γ-HCH	58-89-9	N.D	0.332	0.0184	0.12	5.55
PCB 28	7012-37-5	N.D	0.411	0.088	0.19	22.2
Heptachlor	76-44-8	N.D	N.D	N.D	N.D	N.D
PCB 138	35065-28-2	N.D	N.D	N.D	N.D	N.D
PCB 153	35065-27-1	N.D	N.D	N.D	N.D	N.D
PCB 52	35693-99-3	N.D	N.D	N.D	N.D	N.D
Aldrin	309-00-2	N.D	3.73	0.207	0.90	5.55
Dieldrin	60-57-1	N.D	4.86	0.270	1.11	5.55
Endrin	72-20-8	N.D	6.15	0.342	1.44	5.55
Heptachlor epoxide	1024-57-3	N.D	N.D	N.D	N.D	N.D
p,p_-DDE	72-55-9	N.D	N.D	N.D	N.D	N.D
PCB 118	31508-00-6	N.D	N.D	N.D	N.D	N.D
PCB 101	37680-73-2	N.D	N.D	N.D	N.D	N.D
o,p_-DDT	789-02-6	N.D	N.D	N.D	N.D	N.D
p,p_-DDD	72-54-8	N.D	N.D	N.D	N.D	N.D
p,p_-DDT	50-29-3	N.D	N.D	N.D	N.D	N.D
PCB 180	35065-29-3	N.D	N.D	N.D	N.D	N.D
∑OCPs		N.D	20.2	4.06	7.13	33.3
∑PCBs		N.D	0.411	0.0882	0.22	22.2

Notes: “ND” not detectable.

Tai Wang et al. conducted a survey on the pollution by the dissolved PCBs and OCPs in the surface water of Haihe River and Bohai Gulf in summer, which indicated that the contents of PCBs, HCHs and DDTs in the surface water of both were 0.06 - 3.11 µg/L, 0.05 - 1.07 µg/L and 0.01 - 0.15 µg/L respectively [19]. The industrial wastewater discharged to the trunk stream of Haihe River was probably the major source of PCBs and OCPs in Bohai Gulf. A research done by Minqiao Li et al [20]. suggested that the concentration of PCBs in the East China Sea ranged from 0.59 to 1.68 ng/L, slightly higher than the finding of this study. However, compared with those in other seas at home and abroad, this was still a medium or low level. The research findings showed that there was a significant negative correlation between the concentration of PCB in seawater and the salinity, meaning that the PCB carried by rivers was the major source of PCB in the East China Sea. In the offshore waters of East China Sea, the hexachlorobenzene pollution was slightly higher than the theoretical concentration value. This potential source of pollution may be associated with the e-waste disposal plants along the East China Sea [20].

In most places, industrial HCH (60-70% α-HCH, 5-12% β-HCH and 10-12% γ-HCH), instead of pure lindane (γ-HCH), was used. The ratio between α-HCH and γ-HCH (lindane) was 3-7. In case of the input of lindane, this ratio may approximate or be less than 1. If this ratio went higher than 7, it was probably caused by the long-distance transmission of HCH or the degradation of industrial HCH. The half-life period of α-HCH was longer than that of lindane by 25%, so the higher the ratio was, the longer the transmission distance would be, or the longer time the degradation would take [21].

This study found that OCPs residues were detected in the water at one-third of the 18 sampling sites. Five of these sites were detected to contain α-HCH, four contain β-HCH, three of them contain δ-HCH, only one contain γ-HCH, three contain α-HCH, β-HCH and δ-HCH simultaneously, and one contain α-HCH and γ-HCH simultaneously, with the concentration ratio between the two being 16:7. In the remaining 12 sampling sites, neither α-HCH nor γ-HCH was detected, indicating that the HCH pollution in the Irtysh River was mainly caused by long-distance transmission or degradation of industrial HCH. Judging from the sampling site and the pollution feature, we could find out that the OCPs and HCHs levels were higher at the upper reaches than at lower reaches, and were higher in the tributaries than in the trunk stream, on the whole. DDTs were not detected. As for PCBs, PCB 28 was detected only at the middle and lower reaches. Chen et al. analyzed Yellow River water samples and identified up to 33 PCB homologs with an average concentration of 0.232 ng/L and an average OCP concentration of 8.287 ng/L. The residue concentrations of PCBs and OCPs were highest in the downstream, followed by the upstream, and lowest in the midstream [22]. Sohail et al. analyzed OCPs and PCBs across the entire Indus River Basin and found that the concentrations of ∑OCPs and ∑PCBs ranged from 34 to 1600 pg/L and 3 to 230 pg/L, respectively. Spatial variations in ∑OCPs (p<0.05) showed that the highest levels were in alluvial riverine zone, followed by frozen mountain zone, low-lying zone, and wet mountain zone [23]. From these findings, we could find out that the PCBs concentration in the Irtysh River was almost as high as that in the Yellow River and the Indus, while the OCPs concentration was lower than that in the Yellow River but higher than that in the Indus. The spatial distribution pattern of these pollutants in the Irtysh River was contrary to that of the Yellow River.

Based on the test results, the sampling sites with no OCPs and PCBs detected and the POPs components were rejected. A correlation analysis was made on the samples from 10 of the 18 sampling sites and on the contents of POPs of 8 components (Table 4). The analysis showed that there was certain correlation between the components of POPs in the Irtysh River. Among the 8 components analyzed, 4 exhibited significant positive correlation between them (P < 0.01), and 1 exhibited significant negative correlation (P < 0.05). α-HCH was in extremely significant positive correlation with δ-HCH and β-HCH (P < 0.01), δ-HCH was in extremely significant positive correlation with β-HCH (P < 0.01), Aldrin was in extremely significant positive correlation with Dieldrin and Endrin (P < 0.01), Dieldrin was in extremely significant positive correlation with Endrin (P < 0.01). α-HCH was in significant negative correlation with PCB 28 (P < 0.05).

**Table 4:** Correlation Coefficient Matrix for POPs.

POPs	α-HCH	δ-HCH	β-HCH	γ-HCH	PCB 28	Aldrin	Dieldrin	Endrin
α-HCH	1	0.803^⁎⁎^	0.947^⁎⁎^	0.309	-0.728*	-0.309	-0.309	-0.309
δ-HCH		1	0.848^⁎⁎^	-0.214	-0.504	-0.214	-0.214	-0.214
β-HCH			1	0.327	-0.615	-0.261	-0.261	-0.261
γ-HCH				1	-0.261	-0.111	-0.111	-0.111
PCB 28					1	-0.261	-0.261	-0.261
Aldrin						1	1.000^⁎⁎^	1.000^⁎⁎^
Dieldrin							1	1.000^⁎⁎^
Endrin								1

Notes: *. Correlation is significant at the 0.05 level (single-tailed); **. Correlation is significant at the 0.01 level (two-tailed).

**Fig. 3:**
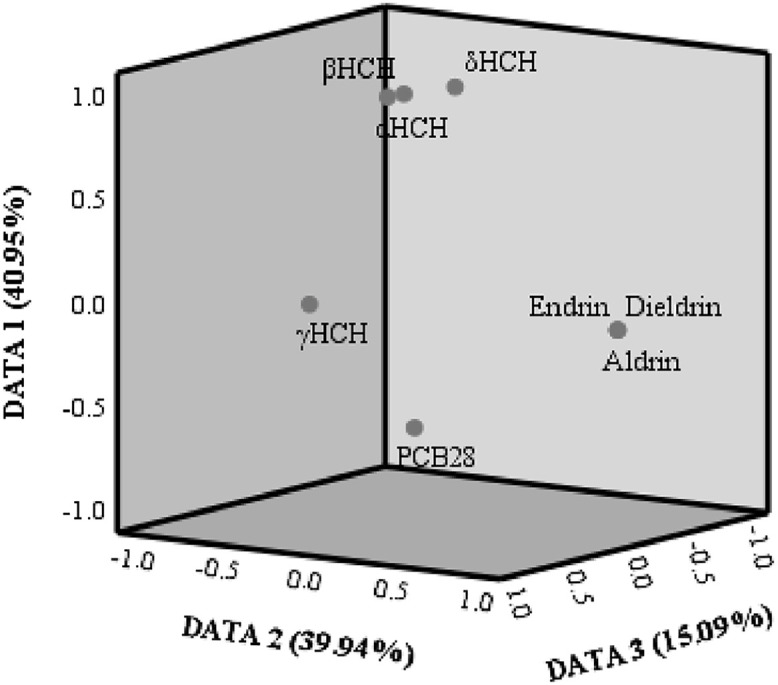
Three-Dimensional Analysis Results for Principal Components.

The principal component analysis was used to analyze the principal components of the 8 components of target compounds, and to study the common relation between relevant variables. The first to the third principal components respectively explained 40.95%, 39.94% and 15.90% of the total variance, thus making the total variance explained come to 95.98%. The load of principal component was as shown in Fig. 3. α-HCH, δ-HCH and β-HCH had a high load on the first principal component, while Aldrin, Dieldrin and Endrin had a high load on the second principal component. The first principal component displayed the HCH residues. However, due to a high content and slow degradation of β-HCH contained in industrial HCH and a low content of γ-HCH, this principal component could still attribute to the use of industrial HCH. The components that had a high load on the second principal component were primarily of OCPs. It was a group of components with similar structure. This principal component could reflect some features of local pest attack. Historically, PCBs and OCPs from industrial and agricultural activities along the Irtysh River have entered the aquatic environment, where they can bind with dissolved organic matter (DOM) and settle into sediments. Some of these pollutants can be released back into the water phase through biological, chemical, and biochemical processes, leading to their detection. To get the detailed information, further investigation was required.

As stipulated in China's *Environmental Quality Standards for Surface Water GB 3838-2002*, the allowable standards for PCBs, HCHs and DDTs were 20 ng/L, 2000 ng/L and 1000 ng/L [24]. The 98/83/EEC ordinances on domestic drinking water established by the Council of the European Community specified that the limits for Aldrin and Dieldrin were 30 ng/L [25]. By referring to the limits for Dieldrin, the limits for Endrin were fixed at 30 ng/L. The levels of OCPs and PCBs at all sampling sites of the Irtysh River did not exceed the relevant stipulated standards. Currently, the evaluation on the ecological risks of OCPs and PCBs mainly focused on the shallow sediments and aquatic products. There was still a lack of researches on the ecological risks in natural waters [[26], [27], [28]].

**Fig. 4:**
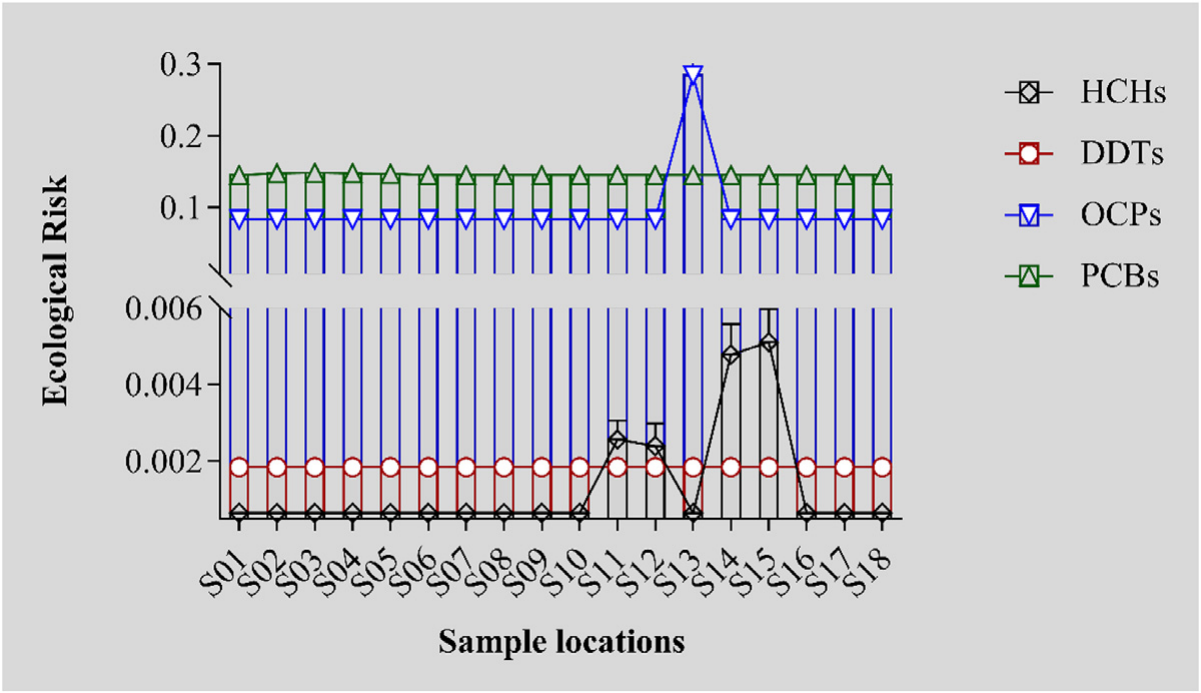
Results of Ecological Risk Evaluation for the Irtysh River.

Based on the standards for three types of surface water in our country and the 98/83/EEC ordinances on domestic drinking water established by the European Union, the target compounds were classified into OCPs, DDTs, HCHs and PCBs, including 6 OCPs, 4 DDTs, 4 HCHs and 7 PCBs. The ecological risk evaluation formula was used to work out the ecological risks of various pollutants on the Irtysh River. The risk quotients of the pollutants contained in all sampling sites of the Irtysh River were shown in Fig. 4. As the quotients of all medium risks at all sampling sites were less than 1, it meant that the organochlorine pollution temporarily had no ecological risks on the Irtysh River.

The peak value of OCPs ecological risk came from S13 at the upper reaches of the Irtysh River, a point near the Irtysh River and Fuyun County. Accordingly, HCHs residues were found at the section of Irtysh River near S13, a result probably caused by the heavy use of pesticides in the past. Though the production and use of most OCPs were prohibited or restricted in many countries, their residues or metabolites could still be detected in various environmental carriers in different regions, owing to their half-life periods and strong mobility [[29], [30], [31]].

## CRediT authorship contribution statement

**Shi-Zhan Tang:** Conceptualization, Methodology, Software, Data curation, Validation, Writing – original draft, Writing – review & editing. **Zhong-Xiang Chen:** Visualization, Investigation, Writing – review & editing. **Qi-Rui Hao:** Supervision. **Yao-Peng Hu:** Supervision. **Ji-Long Wang:** Software, Validation. **Dong-Li Qin:** Supervision. **Peng Wang:** Software, Validation. **Hai-Tao Wang:** Writing – original draft, Data curation, Validation, Writing – review & editing.

## Declaration of competing interest

The authors declare that they have no known competing financial interests or personal relationships that could have appeared to influence the work reported in this paper.

## Data Availability

Data will be made available on request.

## Appendix Supplementary materials

Image, application 1
