# Supplementary material for: Detection of PCBs and OCPs in the Irtysh River Water (GC-MS/MS) and ecological risk assessment
Source: MethodsX. 2024 Sep 2;13:102944. doi: 10.1016/j.mex.2024.102944 (PMC11417519; doi:10.1016/j.mex.2024.102944)
Supplement: Supplementary file 1 [file mmc1.docx]

Supplementary material

| **Number** | **Abbreviation** | **CAS** | **CAS Name** |
| --- | --- | --- | --- |
| 1 | α-HCH | 319-84-6 | Alpha-Hexachlorocyclohexane |
| 2 | Hexachlorobenzene | 118-74-1 | 1,2,3,4,5,6-Hexachlorobenzene |
| 3 | β-HCH | 319-85-7 | Beta-Hexachlorocyclohexane |
| 4 | γ-HCH | 58-89-9 | Gamma-hexachlorocyclohexane |
| 5 | δ-HCH | 319-86-8 | Delta-Hexachlorocyclohexane |
| 6 | PCB 28 | 7012-37-5 | 2,4,4′-Trichlorobiphenyl |
| 7 | Heptachlor | 76-44-8 | 1H-1,4,5,6,7,8,8-Heptachloro-3a,4,7,7a-tetrahydro-4,7-methanoindene |
| 8 | PCB 52 | 35693-99-3 | 2,2′,5,5′-Tetrachlorobiphenyl |
| 9 | Aldrin | 309-00-2 | 1,2,3,4,10,10-Hexachloro-1,4,4a,5,8,8a-hexahydro-1,4:5,8-dimethanonaphthalene |
| 10 | Heptachlor epoxide | 1024-57-3 | 1,4,5,6,7,8,8-heptachloro-2,3-epoxy-3a,4,7,7a-tetrahydro-7-methanoindan |
| 11 | PCB-101 | 37680-73-2 | 2,2′,4,5,5′-Pentachlorobiphenyl |
| 12 | p,p_-DDE | 72-55-9 | 1,1-Dichloro-2,2-bis(p-chlorophenyl)ethylene |
| 13 | Dieldrin | 60-57-1 | 1,2,3,4,10,10-Hexachloro-6,7-epoxy-1,4,4a,5,8,8a-hexahydro-1,4-endo-5,8-dimethanonaphthalene |
| 14 | Endrin | 72-20-8 | 1,2,3,4,10,10-Hexachloro-6,7-epoxy-1,4,4a,5,8,8a-hexahydro-1,4-endo-5,8-dimethanonaph |
| 15 | PCB-118 | 31508-00-6 | 2,3′,4,4′,5-Pentachlorobiphenyl |
| 16 | p,p_-DDD | 72-54-8 | 1,1-Dichloro-2,2-bis(p-chlorophenyl)ethane |
| 17 | o,p_-DDT | 789-02-6 | 1,1,1-Trichloro-2,2-bis(4-chlorophenyl)ethane |
| 18 | PCB 153 | 35065-27-1 | 2,2′,4,4′,5,5′-Hexachlorobiphenyl |
| 19 | p,p_-DDT | 50-29-3 | 1,1,1-Trichloro-2,2-bis(p-chlorophenyl)ethane |
| 20 | PCB 138 | 35065-28-2 | 2,2′,3,4,4′,5′-Hexachlorobiphenyl |
| 21 | PCB-180 | 35065-29-3 | 2,2′,3,4,4′,5,5′-Heptachlorobiphenyl |

Table S1 List of detected PCBs, and OCPs with abbreviations
